# Diagnostic accuracy of the Xpert MTB/Rif Ultra for tuberculosis adenitis

**DOI:** 10.1186/s12879-019-4749-x

**Published:** 2020-01-13

**Authors:** Katherine Antel, Jenna Oosthuizen, Francois Malherbe, Vernon J. Louw, Mark P. Nicol, Gary Maartens, Estelle Verburgh

**Affiliations:** 1 Division of Haematology, Department of Medicine, University of Cape Town, Anzio Rd, Observatory, Cape Town, 7925 South Africa; 2 Department of Surgery, University of Cape Town, Cape Town, South Africa; 3 Division of Medical Microbiology, University of Cape Town, Cape Town, South Africa; 4 Division of Clinical Pharmacology, Department of Medicine, University of Cape Town, Cape Town, South Africa

**Keywords:** Tuberculosis, Xpert MTB/RIF Ultra, HIV, Fine-needle aspiration, Core needle biopsy

## Abstract

**Background:**

The WHO recently recommended the new Xpert MTB/RIF Ultra assay (Ultra) instead of the Xpert MTB/RIF assay because Ultra has improved sensitivity. We report the diagnostic accuracy of Ultra for tuberculous adenitis in a tuberculosis and HIV endemic setting.

**Methods:**

We obtained fine-needle aspirates (FNA) and lymph node tissue by core-needle biopsy in adult patients with peripheral lymphadenopathy of >20 mm. Ultra and mycobacterial culture were performed on FNA and tissue specimens, with histological examination of tissue specimens. We assessed the diagnostic accuracy of Ultra against a composite reference standard of ‘definite tuberculosis’ (microbiological criteria) or ‘probable tuberculosis’ (histological and clinical criteria).

**Results:**

We prospectively evaluated 99 participants of whom 50 were HIV positive: 21 had ‘definite tuberculosis’, 15 ‘probable tuberculosis’ and 63 did not have tuberculosis (of whom 38% had lymphoma and 19% disseminated malignancy). Using the composite reference standard the Ultra sensitivity on FNA was 70% (95% CI 51–85; 21 of 30), and on tissue was 67% (45–84; 16/24) these were far superior to the detection of acid-fast bacilli on an FNA (26%; 7/27); AFB on tissue (33%; 8/24); or tissue culture (39%; 9/23). The detection of granulomas on histology had high senstivity (83%) but the lowest specficity. When compared with culture the Ultra on FNA had a sensitvity of 78% (40-97; 7/9) and tissue 90% (55-100; 9/10).

**Conclusions:**

Ultra performed on FNA or tissue of a lymph node had good sensitivity and high specificity. Ultra had a higher yield than culture and has the advantage of being a rapid test. Ultra on FNA would be an appropriate initial investigation for lymphadenopathy in tuberculosis endemic areas followed by a core biopsy for histopathology with a repeat Ultra on tissue if granulomas are present.

## Background

Lymph nodes and pleura are the most common sites of involvement in extrapulmonary tuberculosis (EPTB), which is more common in people living with HIV (PLWH) [1]. EPTB is difficult to diagnose because it is usually paucibacillary and conventional methods for diagnosis, such as microscopy and culture, have low yields, culture results may take several weeks and histological findings (e.g. granulomas) are not specific for tuberculosis. Following a systematic review, the WHO has recommended that the rapid nucleic acid amplification test Xpert MTB/RIF assay (Xpert) should be used in the diagnosis of EPTB, including lymph node tissue [2]. In this meta-analysis (13 studies, 955 samples), the pooled sensitivity of Xpert on lymph node (tissue or fine needle aspirate (FNA)) was 83.1% (95% CI 72–91), and pooled specificity was 94% (95% CI 88–97%). Xpert has been recently superseded by the Xpert MTB/RIF Ultra assay (Ultra), which has greater sensitivity for the detection of *Mycobacterium tuberculosis* complex in sputum specimens but has lower specificity, especially in patients with previous tuberculosis [3].

The Ultra differs from the earlier Xpert assay in a number of ways: two different multicopy amplification targets (*IS*6110 and *IS*1081) have been added to improve detection of *M. tuberculosis* and melting temperature-based polymerase chain reaction (PCR) analysis (melting curve analysis) is used to improve the detection of rifampicin resistance [4]. Because the limit of detection of Ultra is lower (15.6 bacterial colony-forming units (CFU) per ml compared with 114 CFU per ml with Xpert), the greatest improvement in detection is likely to be in paucibacillary samples. This has been demonstrated in cerebrospinal fluid, where Ultra has much higher sensitivity (95, 95% CI 77–99), versus Xpert at 45%,) with only a marginally reduced specificity (95.6% vs 98.3%) which was thought to be due to antecedent tuberculosis infection [5]. The performance on Ultra in lymph nodes has only been tested in one small retrospective study on 10 frozen samples where it showed an improvement of 50% compared with Xpert, (5/10 samples that were Xpert negative and culture positive were positive on Ultra) [6].

In this study, we determined the diagnostic accuracy of Ultra for the detection of *M. tuberculosis* in specimens of lymph node obtained by FNA and core-needle biopsy.

## Methods

### Study design and participants

We conducted a prospective diagnostic accuracy study of Ultra on both FNA and lymph node core-needle biopsy tissue in patients with suspected tuberculosis adenitis. The study was performed at Groote Schuur Hospital, a tertiary referral academic centre in Cape Town, South Africa. Eligible study participants were adults (≥18 years), both in- and outpatients, referred with enlarged lymph nodes of >20 mm in the widest diameter located in either the cervical, axillary or inguinal region. Patients on tuberculosis therapy were enrolled provided this had been given for <1 month (sub-analyses were done in patients on tuberculosis therapy for <24 h). Patients with contraindications to core-needle biopsy (low platelets, other coagulopathy and bleeding risk, clinically unstable, site of biopsy unsafe) were excluded. Written informed consent was obtained from all participants. The Human Research Ethics Committee of the Faculty of Health Sciences, University of Cape Town, approved the study.

Patients came from within Groote Schuur and from secondary level hospitals and day clinics in the referral area. Results of prior tuberculosis investigations (sputum Xpert or tuberculosis culture from any site within 3 months of referral, or urine lipoarabinomannan (LAM)) were recorded. Details of HIV status, tuberculosis treatment and ART were obtained.

### Data collection

Demographic information, symptoms, symptom duration, HIV test result, and other TB investigations performed were recorded at enrolment. Performance status was graded according to the Eastern European Cooperative Group (ECOG) [7]. The site of biopsy was recorded, along with other sites of lymphadenopathy. The presence and duration of constitutional symptoms (cough, loss of weight night sweats) were specifically enquired about as was the duration that the patient had noted the lymphadenopathy. Blood was taken for a full blood count with differential, lactate dehydrogenase and HIV status and, if positive, a CD4 count and a viral load for those on ART.

### Study procedures and specimen collection

**Fig. 1:**
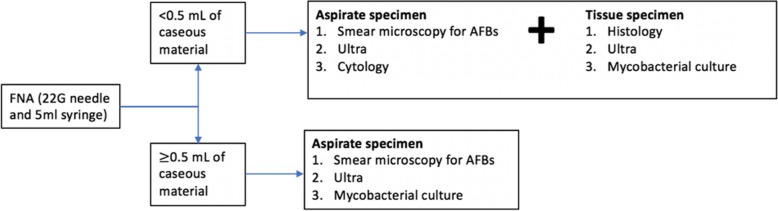
Study procedures

FNA was performed using a 22G needle and 5 mL syringe; further study procedures were determined by the volume of the aspirate sample obtained as shown in Fig. 1. A core-needle biopsy was only performed when < 0.5 mL of caseous material was obtained by the aspirate, due to the risk of causing a draining sinus. Initially, when both an FNA and a biopsy were performed on a participant the Ultra was performed only on the tissue, but there was a protocol change after 25 patients and Ultra was performed on both the FNA and tissue on the same patient. When a patient had both an FNA and a core-needle biopsy the TB culture was only performed on the tissue specimen. For the Ultra on the FNA, the needle and syringe were flushed into a sterile container containing 2 mL of saline. An air-dried smear for AFBs was made at the bedside from a second FNA. For culture from the FNA, the aspirate was flushed directed into mycobacterium culture medium (Middlebrook 7H9 broth medium). The core-needle biopsy was performed by an automated biopsy gun (BARD ﻿Magnum™, CR Bard Inc., Covington, GA, USA) with a 14G needle. If the lymph node was not obviously palpable the biopsy was performed under ultrasound guidance. Two or three cores were sent in formalin for histology (10–15 mm long), an additional core was cut in two with a sterile blade and sent for culture and Ultra, both in 2 ml of 0.9% saline. If all of the tests performed were inconclusive, the patient underwent a repeat core-needle biopsy or an excision biopsy at the discretion of the treating clinician.

### Laboratory tests

FNA and tissue specimens were transported within 2 h of collection to a centralized laboratory and processed individually using standardised protocols by trained laboratory staff. The smear slide made at the bedside was examined by Ziehl-Neelsen (ZN) for AFBs. The tissue obtained by core-needle biopsy was crushed using pestle and mortar. A small portion was smeared on a slide and examined with a ZN stain for AFBs. Mycobacterial culture was performed using an automated liquid mycobacterial culture system (BACTEC™ MGIT™ 960; Becton, Dickinson and Company, New Jersey, USA). Lymph nodes are considered a sterile site and decontamination was not performed prior to liquid biopsy. If the MGIT flagged positive, one droplet was inoculated on 2% blood agar and incubated for 24 h to check for bacterial growth. If there was no bacterial growth, the MGIT was reported as positive for mycobacterium, the specimen was decontaminated with sodium hydroxide (1%) and N-acety-L-cysteine and then repeat MGIT was performed. Positive isolates from culture media were identified by acid-fast staining followed by MRTBDR*plus* testing (Hain LifeScience, Hehren, Germany) to confirm the presence of *M. tuberculosis* and rifampicin and isoniazid sensitivity.

For the Ultra, 1.4 mL of specimen reagent was added to 0.7 mL of aspirate or crushed tissue sample. Results were reported as: invalid (no internal assay control detected); not detected; or detected (with semi-quantitation: trace, very low, low, medium, or high) and rifampicin resistance (detected, not detected, or indeterminate). Staff performing Ultra were blinded to clinical and other microbiological results.

Histological review was performed by a qualified anatomical pathologist who was blinded to the results of the ULTRA and did not have access to the culture, but would have been able to see the results of AFBs on microscopy. If granulomas were identified, a separate ZN stain was performed by the pathologist and a Periodic acid–Schiff (PAS) stains were performed for fungi.

### Case definitions and statistical analysis

We assigned participants to one of three diagnostic categories on the basis of clinical, histological and microbiological investigations. *Definite tuberculosis* (culture-positive on FNA/ tissue for *M. tuberculosis* OR AFBs identified on FNA/tissue) *Probable tuberculosis* (no other diagnosis that would account for lymphadenopathy with one or more of: macroscopic caseation on FNA/tissue OR tuberculosis confirmed microbiologically [Ultra or culture] at a site other than the lymph node (eg pulmonary) OR granulomas on FNA/tissue). The third category was *not tuberculosis (did not fulfil the criteria for the other two categories)*. The Ultra diagnostic accuracy was also reported separately using positive culture only as the reference standard.

Sample size estimation in diagnostic accuracy studies depends on the prevalence of disease [8]. We were expecting a high prevalence of tuberculosis based on a pilot study we conducted of core-needle biopsy in HIV-positive patients, 92% of whom had tuberculosis lymphadenitis [9], but the proportion of patients with tuberculosis in our study was lower than expected (40%) in a pilot phase of our study. We estimated the sensitivity and specificity of Ultra would both be around 90% based on the Cochrane meta-analysis [9]. With a 40% prevalence of tuberculosis a sample size of 87 is needed for 95% CI widths of 10% and with sensitivity and specificity of 90% [8]. We inflated the sample size to 100 because of uncertainties about the diagnostic accuracy of Ultra.

We calculated sensitivity, specificity, negative predictive value (NPV), positive predictive value (PPV) and likelihood rations by defining true or false positives and true or false negatives against the composite reference standard of *probable* or *definite tuberculosis* for our primary analysis. As secondary analyses we also determined the accuracy of Ultra using mycobacterial culture alone as the reference standard. Data was entered into a REDCap® database and analysed using the STATAv14 software package (StataCorp, College Station, Texas, USA). Baseline clinical characteristics were compared using the chi-squared or Fisher’s exact test for categorical variables and the Kruskal-Wallis test for continuous variables. We counted invalid tests (i.e. Ultra-error) as negative results. This study is reported in accordance with the Standards for Reporting of Diagnostic Accuracy Studies Guidelines [10].

### Role of the funding source

The funders had no role in study design, data collection, data analysis, data interpretation, or writing of the report. The corresponding author had full access to all the data in the study and had final responsibility for the decision to submit for publication.

## Results

### Demographic and clinical characteristics

**Fig. 2:**
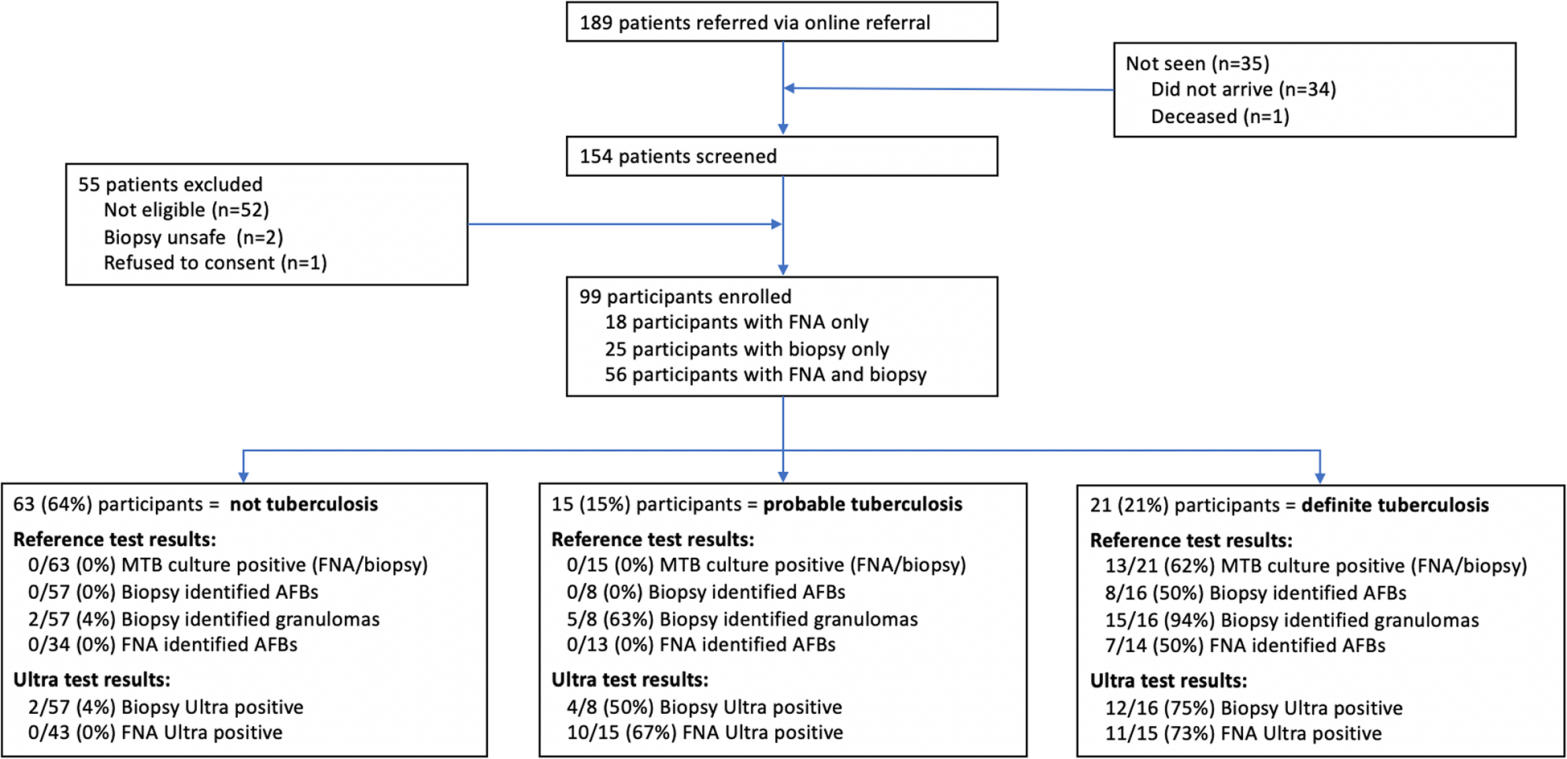
Trial profile. MTB = *Mycobacterium tuberculosis.* AFB = acid fast bacilli. FNA = fine-needle aspirate

Between November 2017 and October 2018, 154 consecutive patients were evaluated for suspected lymphadenopathy and we enrolled 99 participants for the study (Fig. 2). Ultra of both FNA and core-needle biopsy were performed in 56 participants, 25 participants had Ultra testing of a core-needle biopsy only (prior to protocol change), and 18 had Ultra testing of an FNA only. The majority of participants (84%) were seen as outpatients. Of the 51% (*n* = 50) participants who were HIV positive, 62% (*n* = 31) were on ART and had a median CD4 count of 216 cells/mm^3^ (IQR 82–361) and in the participants on ART, 42% (13/31) were virally suppressed with a viral load lower than the detectable limit (<20 RNA copies/mL). The final diagnosis was *definite tuberculosis* in 21 (21%), probable *tuberculosis* in 15 (15%) and *not tuberculosis* in 63 (64%. Table 1 shows baseline characteristics of the participants by diagnostic group.

**Table 1:** Baseline characteristics by diagnostic group

	Total (*n* = 99)	Definite TB (*n* = 21)	Probable TB (*n* = 15)	Not TB (*n* = 63)	*P*-value
Sex					
Males	45 (45%)	9 (43%)	4 (27%)	32 (51%)	0.233
Females	54 (55%)	12 (57%)	11 (73%)	31 (49%)	
Age (median [IQR])	37 (30–49)	32 (28–35)	36 (31–41)	44 (32–57)	**0.003**
HIV status					
Positive	50 (51%)	13 (62%)	8 (53%)	29 (46%)	0.442
Negative	49 (49%)	8 (38%)	7 (47%)	34 (54%)	
On ARVS	31 (62%)	8 (62%)	7 (88%)	16 (55%)	0.263
CD4 count (cells/mm^3^, median [IQR])	216 (82–361)	100 (55–254)	351 (88–497)	252 (92–361)	0.106
< 100	16 (32%)	6 (46%)	2 (25%)	8 (28%)	0.675
100–249	10 (20%)	3 (23%)	1 (13%)	6 (21%)	
≥ 250	24 (48%)	4 (31%)	5 (63%)	15 (52%)	
Performance Score					
ECOG 0	49 (50%)	10 (48%)	9 (60%)	30 (48%)	0.979
ECOG 1	28 (28%)	6 (29%)	5 (33%)	17 (27%)	
ECOG 2	4 (4%)	1 (5%)	0	3 (5%)	
ECOG 3	16 (16%)	4 (19%)	1 (7%)	11 (17%)	
ECOG 4	2 (2%)	0	0	2 (3%)	
Patient Type					
Inpatient	16 (16%)	4 (19%)	2 (13%)	10 (16%)	0.924
Outpatient	83 (84%)	17 (81%)	13 (87%)	53 (84%)	
Previous tuberculosis	24 (24%)	6 (29%)	6 (40%)	12 (19%)	0.205
On tuberculosis treatment	21 (21%)	6 (29%)	6 (40%)	9 (15%)	0.059
Consistency of lymph node					
Firm	81 (87%)	16 (80%)	12 (86%)	53 (90%)	0.491
Fluctuant	12 (13%)	4 (20%)	2 (14%)	6 (10%)	
Symptoms					
Cough	27 (27%)	6 (29%)	3 (20%)	18 (29%)	0.79
Weight loss	47 (47%)	13 (62%)	5 (33%)	29 (46%)	0.222
Night sweats	29 (29%)	8 (38%)	3 (20%)	18 (29%)	0.49
Location of lymph nodes					
Unilateral	61 (62%)	13 (62%)	12 (80%)	36 (57%)	0.262
Bilateral	38 (38%)	8 (38%)	3 (20%)	27 (43%)	
Lymph node FNA/biopsy site					
Neck	85 (86%)	19 (90%)	14 (93%)	52 (83%)	0.881
Axilla	10 (10%)	2 (10%)	1 (7%)	7 (11%)	
Inguinal	4 (4%)	0	0	4 (6%)	
Blood results (median [IQR])					
LDH (units/L)	267 (218–341)	292 (251–341)	218 (194–239)	273 (230–366)	**0.006**
Hb (g/dL)	12.1 (9.4–13.4)	10.5 (8.7–12.1)	11.8 (9.6–12.7)	12.4 (9.7–13.5)	0.041
WCC (cells × 10^9)	6.9 (4.9–9.1)	5.3 (4.0–8.0)	5.7 (4.8–7.3)	7.4 (5.2–9.9)	0.142
Lymphocytes (cells × 10^9)	1.7 (1.0–2.4)	1.2 (0.7–1.9)	1.7 (1.4–2.0)	1.7 (1.0–2.7)	0.197

*P* values <0.05 are shown in bold. Data are n/N; % (95% CI). *ECOG* Eastern Cooperative Oncology Group, *LDH* Lactate Dehydrogenase, *Hb* Haemoglobin, *WCC* White Cell Count

The median age was significantly higher in the *not tuberculosis* group. The groups had similar HIV prevalence and, in those who were HIV positive, similar median CD4 counts. Previous tuberculosis, HIV prevalence and CD4 count was similar in all diagnostic outcome groups. Participants in all groups frequently reported cough, night sweats and weight loss. There was also no significant difference in the clinical examination findings of the lymph node. Lymphocyte count, LDH and total white cell count (WCC) were similar, however, anaemia was more common in the participants with *definite tuberculosis* (present in 81%) compared with the other two groups (*p* = 0.041).

A high proportion of participants had tuberculosis investigations prior to referral and the frequency of positive results were: sputum Xpert 3/22, urinary LAM 1/5, and tuberculosis culture (5/15) (by site: urine 0/1, blood 1/2, sputum 4/12, lymph node 0/1 (tissue)). Chest x-ray had been performed in 36% and reported as ‘suggestive of tuberculosis’ by the referring clinician in 28% of these.

In the *not tuberculosis* group the final diagnosis was: lymphoma in 24 (38%), other malignancy in 19 (30%), reactive lymphadenopathy in 8 (13%), and miscellaneous other causes (enlarged submandibular salivary gland (*n* = 4), bacterial adenitis (*n* = 4), sarcoidosis (*n* = 2), sinus histiocytosis (*n* = 1), branchial cleft cyst (*n* = 1)). The diagnosis of ‘bacterial adenitis’ was based on > 0.5 mL caseous material with lymphadenopathy resolving after drainage and antibiotics, and culture of the caseous material not positive for tuberculosis. In the two cases of sarcoidosis, granulomas were identified on histopathology and chest computed tomography was compatible with sarcoidosis, both participants went on to have a lymph node excision biopsy which was negative on culture for tuberculosis.

### Results of investigations and the performance of the Ultra

Participants underwent a number of different investigations for tuberculosis and histological examination on the lymph node; Fig. 2 shows the participant test results by diagnostic outcome groups. The diagnostic accuracy of the reference tests (i.e. identification of AFBs, culture of lymph node tissue or aspirate) and against the composite reference standard and against culture (of lymph node tissue or FNA) are shown in Table 2. The diagnostic accuracy of Ultra on both FNA and tissue is presented in Table 3 using the composite reference standard, two sub-analyses are presented: the exclusion of participants on tuberculosis therapy, and by using culture (tissue/FNA) alone as the reference test.

**Table 2:** Sensitivity and specificity of each diagnostic test using the composite reference standard and culture of either lymph node tissue or FNA

Tuberculosis test	Sensitivity against composite reference	Specificity against composite reference	Sensitivity against culture	Specificity against culture
AFBs on FNA (*n* = 61)	7/27; 26% (11–46)	34/34; 100% (90–100)	3/8; 38% (9–76)	49/53; 92% (82–98)
FNA culture (*n* = 17)	4/12; 33% (10–65)	5/5; 100% (48–100)	4/4; 100% (40–100)	13/13; 100% (75–100)
AFBs on tissue (*n* = 81)	8/24; 33% (16–55)	57/57; 100% (94–100)	4/10; 40% (12–74)	67/71; 94% (86–98)
Tissue culture (*n* = 75)	9/23; 39% (20–61)	52/52; 100% (93–100)	9/10; 90% (55–100)	65/65; 100% (94–100)
Necrotising granulomas on histology (n = 81)	20/24; 83% (63–95)	55/57; 96% (88–100)	10/10; 100% (69–100)	59/71; 83% (72–91)

Data are n/N; % (95% CI). *FNA* Fine-needle aspirate, *AFBs* Acid fast bacilli, *PPV* Positive predictive value, *NPV* Negative predictive value

**Table 3:** Diagnostic accuracy of Ultra on FNA and tissue measured against the composite reference standard and culture

	Sensitivity	Specificity	PPV	NPV	LR+	LR-
Composite reference standard						
Ultra on FNA (*n* = 73 participants)	21/30; 70% (51–85)	43/43; 100% (92–100)	100%	83%	inv	0.3
Ultra on tissue (*n* = 81 participants)	16/24; 67% (45–84)	55/57; 96% (88–99)	89%	87%	16.8	0.3
Excluding the 21 participants on tuberculosis therapy>24 h						
Ultra on FNA (*n* = 57 participants)	12/20; 60% (36–81)	37/37; 100% (91–100)	100%	82%	inv	0.4
Ultra on tissue (*n* = 66 participants)	10/18; 56% (31–78)	46/48; 96% (86–99)	83%	85%	14	0.5
Culture as the reference standard (*n* = 99 participants)						
Ultra on FNA (*n* = 73 participants)	7/9; 78% (40–97)	50/64; 78% (66–87)	33%	96%	3.5	0.3
Ultra on tissue (*n* = 81 participants)	9/10; 90% (55–100)	62/71; 87% (77–94)	50%	98%	6.9	0.1

Data are n/N; % (95% CI). *FNA* Fine-needle aspirate, *AFBs* Acid fast bacilli, *PPV* Positive predictive value, *NPV* Negative predictive value, *LR* likelihood ratio

**Fig. 3:**
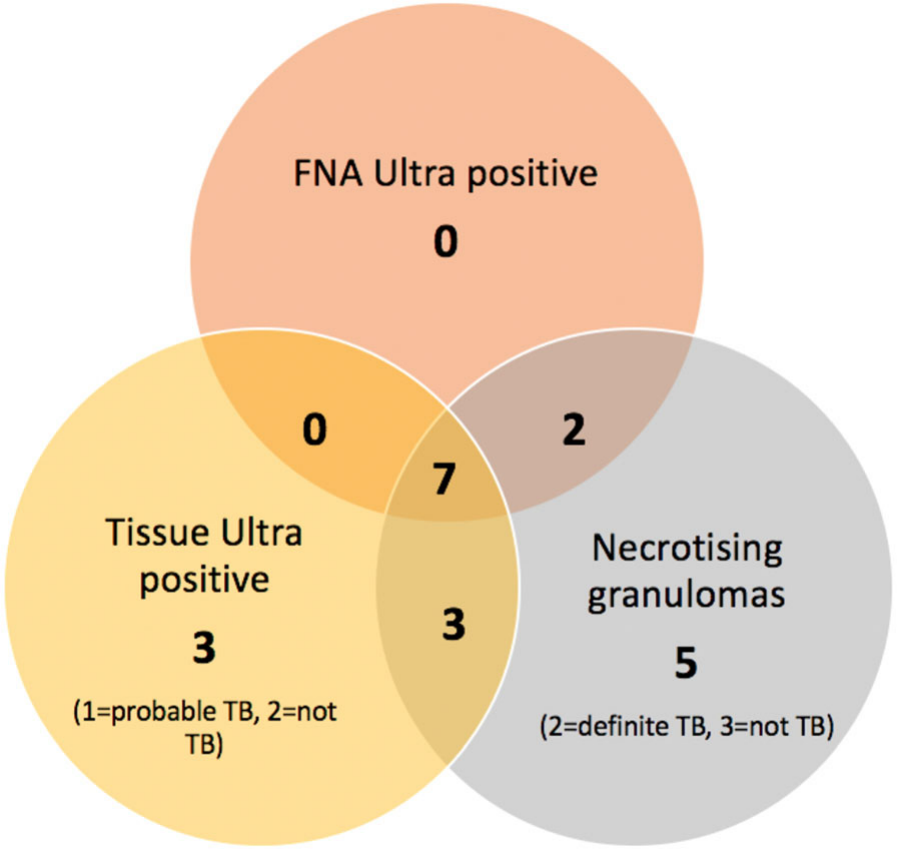
Venn diagram to show the overlap in yield in the tests with the highest yeild for tuberculous adenitis. TB = tuberculosis; FNA = fine-needle aspirate

There were 55 participants who had an FNA and tissue Ultra and histopathological examination of the lymph node. The result concordance is shown in the Venn diagram (Fig. 3). Of the 6 specimens that showed result discordance between FNA (negative) and tissue (positive), all were ‘*trace positive’* on the quantitative result and 2 of these 6 were considered ‘false positives’. These two ‘false positive’ results both had a histologically proven diagnosis of cancer and one of the two had previous tuberculosis 12 years prior (neither were on tuberculosis treatment at the time of biopsy). Although a dual diagnosis of tuberculosis and cancer is possible, histological examination had no features of tuberculosis in these two cases and was considered unlikely. Of the 5 with necrotising granulomas and with a negative Ultra on both FNA and culture, 2 had *definite tuberculosis* (1 culture positive, 1 AFB positive), 1 had *probable tuberculosis* and 2 had sarcoidosis.

There were 4 failed tests (invalid) in the tissue group, and none in the FNA group. There were 12 ‘trace positive’ results, all in the tissue group and 2 of the 12 were considered to be false positive as discussed under discordant results. Rifampicin resistance was identified in 2 of the 39 Ultra positive participants, which was confirmed on line-probe assay.

## Discussion

Our prospective diagnostic accuracy study showed Ultra sensitivity on FNA was 70% (95% CI 51–85; 21 of 30), and on tissue 67% (45–84; 16/24); these were far superior to the detection of acid-fast bacilli on an FNA (26% [11–46; 7/27]), AFB on tissue (33% [16–55; 8/24], FNA culture [33% (10–65; 4/12]) or tissue culture (39% [20–61; 9/23]). The detection of granulomas on histology had high sensitivity (83% [63–95; 20/24]) but had the lowest specificity of all the tests (83% against culture). Our findings support the use of Ultra on FNA as an initial test when tuberculosis adenitis is suspected as Ultra has a high sensitivity, it is minimally invasive, requires no specialised equipment, and gives a rapid result. Where the FNA does not yield a positive result, core-needle biopsy with histopathology and a repeat Ultra on tissue if there are granulomas, would be cost-effective and yield a high diagnostic rate both for tuberculosis and other conditions (notably malignancy).

We had high precision for Ultra specificity, which is a key parameter given that specificity of Ultra was lower than Xpert in sputum [3]. The sensitivity of Ultra on FNA was 67% in our study, which is lower than the 87.6% reported in the Cochrane review [11], but they found sensitivity was lower in adults and with lower prevalence of tuberculosis.

The lower sensitivity of AFB detection and culture on FNA and tissue was poor in our study and is consistent with other studies [12–17]. From our data, the air-dried smear for AFBs from the aspirate can be replaced by the Ultra. Culture is limited both by low sensitivity on the lymph node as well as a long turn-around-time, but it still plays an important confirmatory role in diagnosis particularly where drug resistance is suspected.

There is uncertainty surrounding the interpretation of trace positivity of the Ultra from respiratory samples [3]. In our study, we only had ‘trace positive’ results on the tissue (12) with none on the aspirate. Of the 6 discordant FNA and tissue results, all were negative on aspirate and trace positive on tissue and two of these 6 were false positives. We suggest that trace positive Ultra results from lymph node tissue or FNA be regarded as positive, but that this result is interpreted together with clinical and histopathological findings. Where the histopathological findings do not show necrotizing granulomas; an excision biopsy would be preferred as this scenario is most likely to represent a false positive result.

Our study has several limitations. First, without a perfect reference standard we needed to define a reference standard; we tried to address this by clearly defining ‘definite’ and ‘probable’ tuberculosis. The most stringent definition for a true positive would be culture positivity. We felt it was justifiable to include detection of AFB in the case definition for *definite tuberculosis* because adenitis caused by nontuberculous mycobacteria is rare (typically only seen in non-BCG immunized children [17]) and we know that culture has relatively low sensitivity on lymph nodes, in particular on FNA [18, 19]. Futhermore, in our cohort the culture positive cases were all of m. tuberculosis with no other types of tuberculosis cultured. The problems defining case positivity especially in extra-pulmonary tuberculosis is a familiar challenge, in the Cochrane systematic review of Xpert for extra-pulmonary tuberculosis the specificity of Xpert on FNA was 86% when culture was the reference standard, but in the latent class meta-analysis model with a less stringent reference standard improved to 99%, which is likely to be a much more accurate reflection of specificity [11]. Further impacting culture detection rates in our study is that we included participants on tuberculosis therapy; a sub-analysis was performed excluding these participants (Table 3) where it did not appear to affect the results. Given the low yield for culture, our detection rates might have been higher if we sent both the aspirate and tissue for tuberculosis culture but due to financial constraints, we only sent one specimen for culture (either the aspirate or the tissue).

A major strength of our study is that is a prospective cohort study that tests the use of the Ultra in a ‘real-world’ clinic setting and can therefore address both diagnostic accuracy as well as clinical utility. We included participants in whom the final diagnosis of cervical swelling was not lymphadenopathy; this could be seen a limitation in that in these few situations the material tested was not lymph node tissue (e.g submandibular gland tissue) but there were no false positives in these patients and this answers an important clinical question about the risk of false positive results if tissue other than lymph node is biopsied. Other study strengths include the use of a standard protocol, and a single operator performing the FNA and core-needle biopsy.

## Conclusion

The Ultra on FNA and on tissue had good sensitivity, and high specificity in our primary analysis. Ultra on FNA is a cheap and simple bedside procedure with a fast turnover time and is the most appropriate the initial diagnostic test where tuberculosis adenitis is suspected. AFBs on air-dried smear on FNA has low sensitivity and should be replaced by the Ultra. Core-needle biopsy with a repeat Ultra when granulomas are identified is an appropriate second investigation as it may confirm tuberculosis infection and importantly allows for histological examination and early diagnosis of lymphoma or other malignancies without the need for excision biopsy.

## Data Availability

The datasets used and/or analysed during the current study are available from the corresponding author on reasonable request.

## Abbreviations

AFB: Acid-fast bacilli
ART: Antiretroviral therapy
CI: Confidence Interval
ECOG: Eastern European Cooperative Group
EPTB: Extrapulmonary tuberculosis
FNA: Fine-Needle Aspiration
LAM: Lipoarabinomannan
OR: Odds Ratio
OS: Overall Survival
PLWH: People Living with HIV
Ultra: Xpert MTB/RIF Ultra assay
Xpert: Xpert MTB/Rif Assay
