# Correction to: Diagnostic accuracy of the Xpert MTB/Rif Ultra for tuberculosis adenitis

**DOI:** 10.1186/s12879-020-4917-z

**Published:** 2020-03-02

**Authors:** Katherine Antel, Jenna Oosthuizen, Francois Malherbe, Vernon J. Louw, Mark P. Nicol, Gary Maartens, Estelle Verburgh

**Affiliations:** 1 Division of Haematology, Department of Medicine, University of Cape Town, Anzio Rd, Observatory, Cape Town, 7925 South Africa; 2 Department of Surgery, University of Cape Town, Cape Town, South Africa; 3 Division of Medical Microbiology, University of Cape Town, Cape Town, South Africa; 4 Division of Clinical Pharmacology, Department of Medicine, University of Cape Town, Cape Town, South Africa

**Correction to: BMC Infect Dis**

**https://doi.org/10.1186/s12879-019-4749-x**

After publication of the original article [1], we were notified that there is a mistake in the article note. Vernon J. Louw and Mark P. Nicol have been erroneously marked as equal contributors.

Gary Maartens and Estelle Verburgh have contributed equally to this work.
